# Assessment of China’s food and nutrition policies based on healthy food environment policy index (food-EPI): focusing on governance, funding and resources, platforms for interaction

**DOI:** 10.3389/fnut.2026.1831021

**Published:** 2026-05-19

**Authors:** Man Zhang, Yi Zhang, Zhenhui Li, Suying Chang, Shuyi Zhou, Xing Wang, Mingzhu Zhou, Na Zhang, Guansheng Ma

**Affiliations:** 1 Institute of Food and Nutrition Development, Ministry of Agriculture and Rural Affairs, Beijing, China; 2 Department of Nutrition and Food Hygiene, School of Public Health, Peking University, Beijing, China; 3 Child Health Development Section, United Nations International Children’s Emergency Fund (UNICEF) Office for China, Beijing, China; 4 Ningbo Medical Center Lihuili Hospital, Ningbo, China; 5 Nanchang Municipal Health Commission, Nanchang, China; 6 Laboratory of Toxicological Research and Risk Assessment for Food Safety, Peking University, Beijing, China

**Keywords:** funding and resources, governance, healthy food environment policy index, platforms for interaction, policy

## Abstract

**Background:**

China currently faces the Triple Burden of Malnutrition, which includes persistent undernutrition, micronutrient deficiencies, and overweight/obesity. Although a life-course nutrition policy architecture has been established, implementation remains constrained fragile governance, inadequate funding and resources, and the absence of robust platforms for interaction. A China-adapted Healthy Food Environment Policy Index assessment is urgently needed to systematically benchmark policy comprehensiveness against international best practice and to inform evidence-based governance optimization.

**Methodology:**

This study evaluated three domains of China’s food and nutrition policy—governance (4 indicators), funding and resources (3 indicators), and platforms for interaction (4 indicators)—based on the Food-EPI framework. An expert panel (*n* = 13) conducted quantitative scoring for each indicator, calculating average scores and implementation percentages for both indicators and domains. Policy implementation levels were then categorized accordingly. The evaluation primarily comprised seven steps: finalize the research protocol; collect policy documents; draft evidence document; validate supporting documents; convene an expert panel workshop; make recommendations; and disseminate findings to government, the public, and stakeholders.

**Results:**

A total of 15 relevant policies were primarily included. Overall, China’s food and nutrition policies rated as medium across all three domains—governance, funding and resources, and platforms for interaction. Specific scores and implementation levels were as follows: governance 3.12 ± 0.98 (62.4%), funding and resources 3.23 ± 0.96 (64.6%), and platforms for interaction 3.46 ± 0.90 (69.2%). Among the 11 indicators, 90.9% were rated at a medium level, 9.1% at a high level, with no indicators classified as low or extremely low.

**Conclusion:**

China’s food and nutrition policies have established a foundational basis in infrastructure support, resource allocation, and coordination; however, they remain in a transitional phase from framework development toward high-quality implementation. Future efforts should focus on optimizing resource allocation, strengthening professional support systems, and advancing data integration and sharing to further enhance policy implementation effectiveness.

## Background

**Figure 1:**
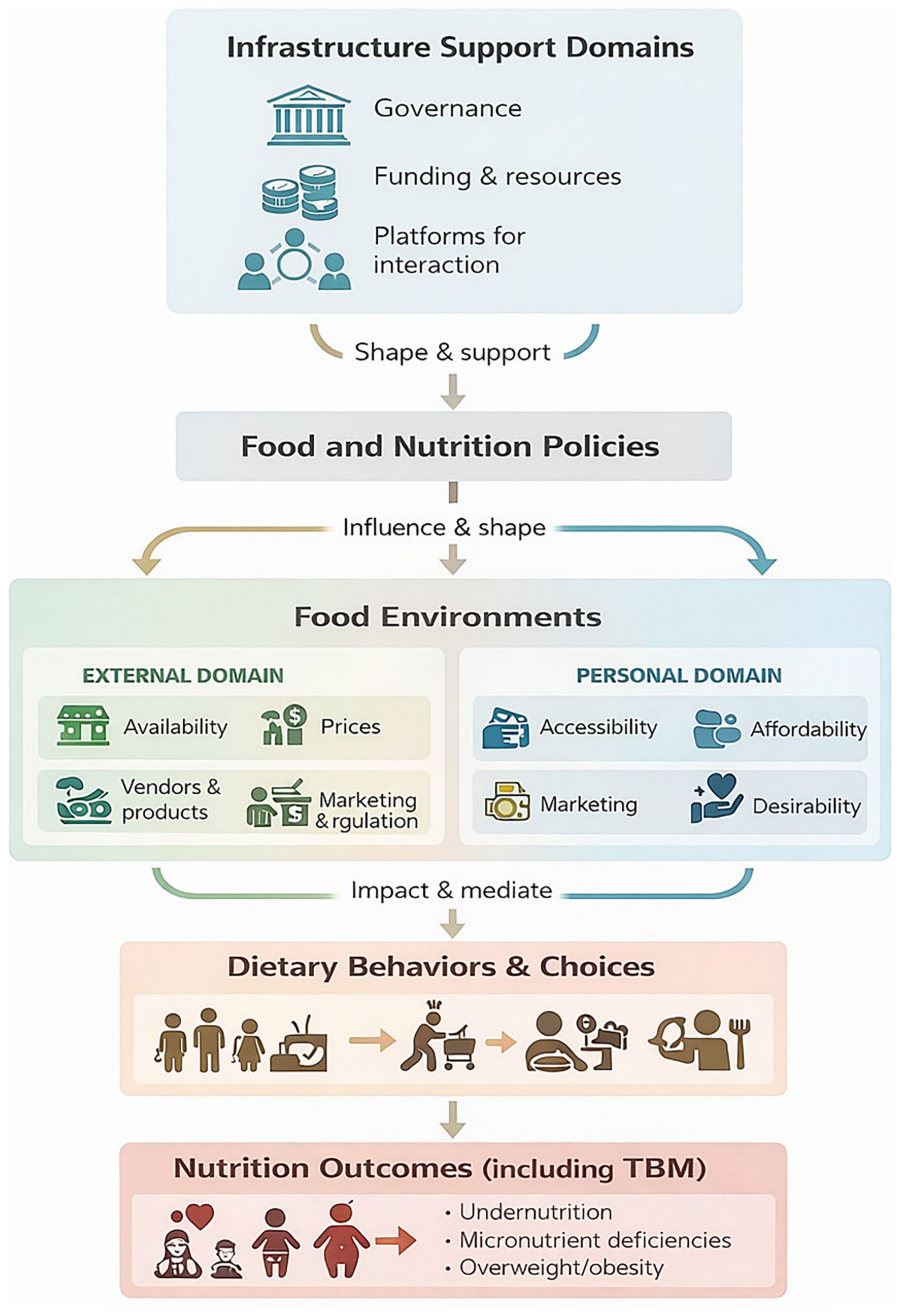
Conceptual framework. This framework illustrates how infrastructure-support domains shape food and nutrition policies, which influence food environments (external and personal), thereby impacting dietary behaviors and contributing to nutrition outcomes, including the triple burden of malnutrition. The framework is adapted from Turner et al. (49) and the UNICEF Innocenti framework on food systems for children and adolescents (50).

The nutritional and health status of a nation’s population serves as a crucial indicator of its level of social development and public health capacity. Currently, China is undergoing a critical phase of nutritional transition, with manifestations of malnutrition becoming increasingly complex. The country now faces the Triple Burden of Malnutrition (TBM), characterized by the coexistence of persistent undernutrition, micronutrient deficiencies, and overweight/obesity (1). Although protein–energy malnutrition has declined markedly, inadequate intakes of vitamin A, iron, zinc and other micronutrients remain widespread. In 2021, the affected population in China reached 146.1 million (2). Concurrently, excessive consumption of oils, salt and sugar, coupled with a fat-energy ratio that exceeds dietary recommendations, has become normative. The resulting surge in overweight and obesity has pushed the combined prevalence among Chinese adults to 50%, according to a cross-sectional study of 15.8 million individuals (3). This dual nutritional transition is driven primarily by rapid changes of the food environment (4), which in turn is highly dependent on policy guidance and regulation. Therefore, establishing evidence-based food and nutrition policies and fostering a healthy food environment have become crucial pathways for promoting healthy lifestyles and alleviating the triple burden of malnutrition (5) (Figure 1). China has consistently prioritized food and nutrition initiatives (6). The report of the 20th National Congress of the Communist Party of China and the 15th Five-Year Plan explicitly advocate advancing the Healthy China initiative, placing safeguarding public health at the forefront of strategic development (7, 8). A series of policies have been enacted, including the National Nutrition Plan (2017–2030) (9), the Healthy China Initiative (2019–2030) (10), and the National Healthy Lifestyle Campaign (11). These policies have progressively established a nutrition and health policy framework covering all populations and the entire life cycle.

However, the continuous refinement of the policy framework imposes higher demands on its implementation. Although China has established a multi-departmental coordination mechanism for nutrition and health led by the National Health Commission and achieved some progress in policy integration, multiple structural challenges persist in practical execution (12–15). Targeted adjustments and optimizations to the existing system are urgently needed based on systematic evaluation. However, existing research has largely focused on evaluating the effectiveness of specific nutritional interventions [such as food nutrition labeling systems (16) and school meal standards (17)], with relatively limited exploration of the institutional and resource foundations essential for policy implementation. The institutional and resource foundations for policy implementation are centered on three interconnected domains. Governance serves as institutional safeguards, establishing transparency, accountability, and public participation mechanisms to foster healthy food environments and reduce health inequalities. Funding and resources involve allocating financial, human, and technical resources to nutrition promotion and chronic disease prevention. Platforms for interaction require establishing collaborative mechanisms across departments, administrative levels, and multiple stakeholders, whose effectiveness directly determines the consistency and implementation efficacy of cross-sectoral food and nutrition policies.

The Healthy Food Environment Policy Index (Food-EPI) is a policy assessment tool proposed in 2013 by the International Network for Food and Obesity/Non-communicable Diseases Research, Monitoring and Action Support (INFORMAS) (18). Following its initial implementation in New Zealand in 2014 (19), it has been adopted in approximately 40 countries worldwide, establishing itself as an international benchmark for evaluating food and nutrition policies (20–27). The tool arrays food and nutrition policies into two dimensions. The infrastructure support dimension includes six domains: leadership, governance, monitoring and intelligence, funding and resources, platforms for interaction, and nutrition and health in all policies. The policies dimension includes seven domains: food composition, food labeling, food promotion, food provision, food retail, food prices, and food trade and investment. Empirical evidence demonstrates that the Food-EPI framework possesses dual functionality: it enables cross-national policy benchmarking while exhibiting contextual adaptability, allowing for localized calibration in accordance with divergent governance architectures, policy priorities, and country-specific nutritional challenges. Policy implementation is evaluated by benchmarking against international best practices. Mexico conducted two policy evaluations using Food-EPI in 2016 and 2024, revealing significant deficiencies in governance structures, funding guarantees, and inter-sectoral collaboration (20). In South Africa, the Food-EPI framework expanded its original indicator system by adding 12 metrics specifically addressing the double burden of malnutrition (DBM). Findings revealed institutional gaps in safeguarding against commercial interference, a lack of dedicated budgets and specialized agencies for nutrition work, and the absence of institutionalized multisectoral coordination mechanisms (21). A comparative Food-EPI study across four South Asian countries (Bangladesh, India, Pakistan, Sri Lanka) further revealed common challenges including insufficient policy transparency, inadequate commercial oversight, and absent multisectoral coordination mechanisms (22). A study applying Food-EPI across 11 countries in six regions further confirmed that most nations demonstrate weaknesses in restricting commercial conflicts of interest and establishing dedicated budgets, with only a few countries achieving high levels on select indicators (26).

Food-EPI has been applied mainly in countries such as New Zealand, Australia, and Mexico, whose institutional settings differ markedly from that of China. In contrast, China’s food and nutrition policy system is characterized by strong government leadership, multi-sectoral coordination, top-down strategic deployment, and hierarchical implementation across central and local levels. In addition, nutrition governance in China is embedded within broader national policy agendas, including the Healthy China Initiative, the food safety governance system, and the comprehensive framework for chronic disease prevention and control, reflecting a high degree of policy integration and administrative mobilization capacity (6). Therefore, directly applying the standard Food-EPI framework may not adequately capture the distinctive features of China’s institutional context or the main structural barriers to policy implementation. Accordingly, this study does not mechanically duplicate the Food-EPI; instead, while adhering to its underlying principles and evaluation procedures, the research focus is adapted to China’s policy context and governance structure. Specifically, recognizing that China’s food and nutrition policy system has taken preliminary shape but that institutional implementation capacity remains a critical constraint on policy effectiveness, this study prioritizes three foundational domains closely tied to implementation capacity: governance, funding and resources, and platforms for interaction. This focus is guided by three considerations. First, these domains capture the most critical institutional determinants of healthy food environment development in China, particularly in terms of government accountability, resource allocation, and cross-sectoral coordination. Second, compared with an inventory of specific policy instruments, assessing underlying system-level support conditions is more consistent with China’s current transition from policy formulation to effective implementation and governance optimization. Third, within China’s government-led and multi-level governance system, foundational support domains largely determine whether nutrition policies can be effectively translated from central decision-making into coordinated sectoral action and local implementation.

Thus, this study employs the Food-EPI framework as a context-adaptable analytical tool to assess the adequacy of China’s food and nutrition policy system across the three domains of governance, funding and resources, and platforms for interaction. By doing so, it aims to identify both the underlying strengths and structural weaknesses in China’s efforts to build a healthy food environment, thereby providing empirical evidence and strategic insights for policy improvement.

## Methods

Building upon the Food-EPI framework, through extensive literature translation, expert consultation, and discussions among project team members, a methodology for assessing China’s food and nutrition policies was established. This methodology encompasses an indicator framework and defined steps.

### Indicator framework

The assessment retains three infrastructure domains of the Food-EPI—governance (4 indicators), funding and resources (3 indicators), and platforms for interaction (4 indicators)—for a total of 11 indicators (Table 1).

**Table 1:** Indicator framework for governance, funding and resources, and platforms for interaction.

Domain	Description of good practice for the domain	Indicators and description of good practice for each indicator
Governance	The government establishes structures to ensure transparency and accountability, and encourages broad participation and inclusivity of communities in policy formulation and implementation.	GOVER1: Robust procedures to restrict commercial influences on the development of policies related to food environments
		GOVER2: Policies and procedures are implemented for using evidence in the development of food policies.
		GOVER3: Policies and procedures are implemented for ensuring transparency in the development of food policies.
		GOVER4: The government ensures access to comprehensive nutrition information and key documents (e.g., budget documents, annual performance reviews and health indicators) for the public.
Funding and Resources	Adequate funding is allocated to population-level nutrition promotion to create healthy food environments, improve nutritional status, and reduce obesity, diet-related chronic non-communicable diseases, and associated inequities.	FUND1: Funding for the promotion of healthy eating and healthy food environments, as a proportion of total health spending and/or in relation to the diet-related NCD burden is sufficient to reduce obesity and diet-related NCDs.
		FUND2: Government funded research is targeted at improving food environments, reducing obesity, NCDs and their related inequalities.
		FUND3: There is a statutory health promotion agency in place that includes an objective to improve population nutrition, with a secure funding stream.
Platforms for Interaction	Governments provide formal platforms and opportunities for line ministries, all levels of government, and non-state actors (NGOs, private sector, academia) to align their actions, ensuring that food-environment, nutrition, and NCD-reduction policies are consistent, efficient, and effective across sectors and jurisdictions.	PLAT1: There are robust coordination mechanisms across departments and levels of government (national and local) to ensure policy coherence, alignment, and integration of food, obesity and diet-related NCD prevention policies across governments.
		PLAT2: There are formal platforms between the government and the commercial food sector to implement healthy food policies.
		PLAT3: There are formal platforms for regular interactions between government, civil society and academia on food policies and other strategies to improve population nutrition.
		PLAT4: The government leads a broad, coherent, effective, integrated, and sustainable systems-based approach with local organizations to improve the healthiness of food environments at a national level.

### Evaluation steps

**Figure 2:**
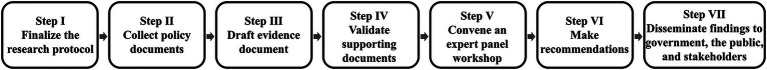
Steps for evaluating China’s food and nutrition policies.

The evaluation of China’s food and nutrition policies primarily involves seven steps (Figure 2).

- Step I: Finalize the research protocol

Translate the Food-EPI into Chinese. The translation was conducted by researchers with overseas academic experience and subsequently validated through expert consultation. Following extensive literature translation, expert review, and discussions among the research team members, the final research protocol was established. On this basis, the research team incorporated the institutional context of China’s food and nutrition policy system and made context-specific adaptations to the relevant formulations of the original Food-EPI framework, ensuring that the indicator definitions are aligned with China’s policy environment while preserving the framework’s core evaluation logic and principles of international benchmarking.

- Step II: Collect policy documents

Collect policies related to China’s healthy food environment governance, funding and resources, and platforms for interaction from August 2019 to June 2024. Policy documents include laws and regulations, action plans, standards, guidelines, and initiatives. Collection was conducted by searching government websites (e.g., China Government Network, National Health Commission official website, Ministry of Agriculture and Rural Affairs official website), academic institution websites (e.g., National Institute of Nutrition and Health official website), NGO websites (e.g., China Cuisine Association, China Food Industry Association official websites), academic search engines (e.g., CNKI, Wanfang Data), and non-academic search engines (e.g., Baidu).

Policy inclusion criteria: Policy documents must be in Chinese, and included policies must be current food and nutrition policies in China (national or local). The policy collection team comprised six researchers, with each indicator independently completed by two researchers.

- Step III: draft evidence document

Following the completion of policy collection, researchers compile a draft evidence document covering existing policies and their implementation status, including enactment dates, monitoring activities, implementation progress, and target achievement rates. The research team developed a preliminary mapping table linking “policy documents” to Food-EPI indicators based on the definitions and descriptions of each indicator. Each policy document was independently reviewed by two researchers to determine whether its content was directly relevant to any specific indicator. If a single policy document addressed multiple indicators, it could be assigned to more than one indicator; however, the specific provisions, implementation measures, or institutional arrangements corresponding to each indicator needed to be clearly specified. In cases where discrepancies arose in the classification results, a third researcher conducted a review, and consensus was reached through group discussion within the research team. The preliminary classification was subsequently subjected to expert consultation during the evidence validation stage to enhance the accuracy and consistency of the classification.

Additionally, the evidence document incorporates examples of international best practices serving as a “benchmark,” based on the document Benchmarking Food Environments 2017: Progress by the New Zealand Government on Implementing Recommended Food Environment Policies & Priority Recommendations compiled by the INFORMAS Secretariat (28). This document was compiled by the INFORMAS Secretariat using the international food policy action database “NOURISHING” developed by the World Cancer Research Fund. The research team updated these best practice examples through literature reviews. The selection of international best practices followed these principles: First, the selected cases must have a clear correspondence with the Food-EPI indicators included in this study. Second, the practices must have been formally implemented by governments at the national or subnational level, rather than remaining at the stage of policy proposals or academic recommendations. Third, the practices should demonstrate well-defined institutional design, implementation mechanisms, or monitoring and evaluation frameworks. Finally, priority was given to policy practices that have been cited as exemplary cases in INFORMAS, the NOURISHING database, or previous Food-EPI studies. The research team initially conducted a preliminary screening based on these databases and published literature, and then updated the cases by incorporating recent international research and policy documents. This process resulted in a list of international best practices, whose applicability and representativeness were further validated through subsequent expert consultation.

- Step IV: validate supporting documents

After completing the draft supporting documents, the research team invited 10 experts and government officials to validate the documents’ completeness and accuracy. Expert consultation meetings were conducted online. The research team held two rounds of consultation meetings until no further revisions were proposed by the experts and government officials. The verification covered two aspects: first, to confirm whether the policy information listed in the evidence document was complete, accurate, and still valid; second, to assess whether the classification of policies under each indicator was appropriate, and whether the international best practice examples corresponded to the relevant indicators. Suggestions from experts regarding additional policies, adjustments to classification, or revisions to international cases were recorded item by item by the research team and incorporated into revisions, resulting in the final version of the evidence document.

- Step V: convene an expert panel workshop

Form an expert panel and invite its members to participate in a workshop to evaluate the policy. Inclusion criteria for the expert panel: individuals with professional experience in food and nutrition policy, representing independent non-governmental organizations or institutions. Prior to the workshop, distribute meeting materials to all experts via email, including a methodological overview, evidence documents, and scoring sheets. Provide evaluative scores and recommendations based on predefined criteria (Table 2). Before scoring each indicator, present experts with examples of relevant policies developed by the national government and international best practices. Subsequently, experts were asked to conduct independent scoring based on the presented evidence. The scoring range was from 1 to 5, indicating the extent to which current policy development and implementation in China align with international best practices. Indicators with large variations in scores were prioritized for in-depth discussion. Where disagreements persisted, consensus was reached through structured discussion. To minimize potential misunderstandings during the scoring process, the research team reiterated the definitions of each indicator, the basis for policy classification, and the correspondence with international best practices prior to scoring, ensuring that all experts conducted their assessments based on a common understanding.

**Table 2:** Scoring criteria.

Score	Criteria	Policy alignment with best practices (Note: The “alignment” column signifies the extent to which domestic policies adhere to recognized international best practices)
1	Absence or nominal policy presence	<20
2	Rudimentary policy structures	20–40%
3	Intermediate policy implementation	40–60%
4	Robust policy strategies	60–80%
5	Comprehensive and holistic policy framework	>80%

The Food-EPI score is calculated based on the expert ratings for each indicator. The specific calculation process is as follows. All calculations were performed using Microsoft Excel (Microsoft Office 2016; Microsoft Corporation, Redmond, WA, USA).

1. Calculate the average score and percentage for each indicator. (a) Indicator Score = $\frac{\sum (\text{Expert Scores})}{\text{Number of Experts}}$ . (b) Indicator Implementation Percentage = $\frac{\text{Indicator Score}}{5}*100\%$ . For example, if an indicator has an score of 3.0, then the indicator implementation percentage is 60%.
2. Calculate the average score and percentage for each domain. Each indicator is weighted at 1. (a) Domain Score = $\frac{\sum (\text{Indicate Scores})}{\text{Number of Indicates}}$ . (b) Domain Implementation Percentage = $\frac{\text{Domain Score}}{5}*100\%$ . For example, if a domain has an score of 3.0, then the domain implementation percentage is 60%.
3. Implementation levels for each indicator and domain were classified as high (≥75%), medium (50–75%), low (25–50%), and very low (<25%). For example, an indicator scored at 60% is rated as medium.

- Step VI: Make recommendations

Based on the scoring results, recommendations are made for the formulation, revision, and improvement of China’s food and nutrition policies, aiming to jointly build a healthy food environment and promote health for all.

- Step VII: Disseminate findings to government, the public, and stakeholders

Feedback including Food-EPI scores and proposed recommendations is provided to government, the public, and other stakeholders to facilitate policy implementation.

## Results

### Characteristics of included policies

This report incorporates 15 key policies or related documents as the basis for subsequent evaluation (Table 3). All included policies are current food and nutrition policies in China. International best practices and local evidence are presented in Supplementary Table S1.

**Table 3:** Characteristics of policies or related documents.

Name	Date of issue	Agency	Scope	Type	Mandatory level	Corresponding indicator
Law of the People’s Republic of China on donations to public welfare causes (29)	June 28, 1999	The standing committee of the national people’s congress	National	Law	High	GOVER1
China’s food and nutrition development outline (2014–2020) (31)	January 28, 2014	The state council	National	Action plan	Medium	GOVER2
Law of the People’s Republic of China on food safety (30)	April 29, 2021	The standing committee of the national people’s congress	National	Law	High	GOVER2 GOVER3
Regulations of the People’s Republic of China on open government information (32)	April 15, 2019	The state council	National	Regulation	High	GOVER4
Sports law of the People’s Republic of China (51)	June 24, 2022	The standing committee of the national people’s congress	National	Law	High	GOVER4
Implementation measures for the improvement plan for rural students in compulsory education (52)	October 31, 2022	The ministry of education and six other departments	National	Regulation	High	FUND1
Law of the People’s Republic of China on the advancement of science and technology (30)	December 24, 2021	The standing committee of the national people’s congress	National	Law	High	FUND2
The medium- and long-term plan for the prevention and treatment of chronic diseases in China (2017–2025) (53)	January 22, 2017	The state council	National	Action plan	Medium	FUND2
Maternal and child health action plan (2018–2020) and healthy children action plan (2018–2020) (54)	April 27, 2018	National health commission	National	Action plan	Medium	FUND2
Basic medical and health care and health promotion law of the People’s Republic of China (55)	December 28, 2019	The standing committee of the national people’s congress	National	Law	High	FUND3
Inter-ministerial joint conference system for the prevention and control of major diseases under the state council (56)	November 19, 2015	The state council	National	Working mechanism	High	PLAT1
Outline for the reform and development of China’s food structure in the 1990s (57)	May 27, 1993	The state council	National	Action plan	Medium	PLAT1
Outline of the healthy China 2030 plan (10)	October 25, 2016	The state council	National	Action plan	Medium	PLAT3
National healthy lifestyle action plan (2017–2025) (58)	April 25, 2017	National health commission	National	Action plan	Medium	PLAT4
National nutrition plan (2017–2030) (9)	June 30, 2017	The state council	National	Action plan	Medium	PLAT4

### Overview of the expert panel

A total of 13 experts participated in the policy scoring for this workshop, with 13 evaluation forms returned, achieving a 100% response rate. The 13 experts represented diverse backgrounds: academic institutions (*n* = 7), national and local Centers for Disease Control and Prevention (*n* = 5), and the food industry (*n* = 1), ensuring strong representativeness. All 13 experts held senior professional titles and possessed over 10 years of experience in their respective fields, conferring high authority. Additionally, 10 experts who had previously participated in supporting document validation were invited to the scoring workshop to ensure continuity and elevate the assessment process through their specialized expertise (Table 4).

**Table 4:** Socio-demographic characteristics of the expert panel.

Characteristics	Group	Number	Percentage
Gender	Male	7	53.8%
	Female	6	46.2%
Field of expertise	Nutrition and food hygiene	4	30.8%
	Public health	2	15.4%
	Marketing	2	15.4%
	Public policy	2	15.4%
	Clinical medicine	1	7.7%
	Government administration	1	7.7%
	Communication studies	1	7.7%
Organization	Higher education institutions	3	23.1%
	National and local centers for disease control and prevention	5	38.4%
	Chinese nutrition society	1	7.7%
	Other research institutions	4	30.8%
Professional title	Senior professional title	9	69.2%
	Associate senior professional title	4	30.8%

### Scores and implementation levels

**Figure 3:**
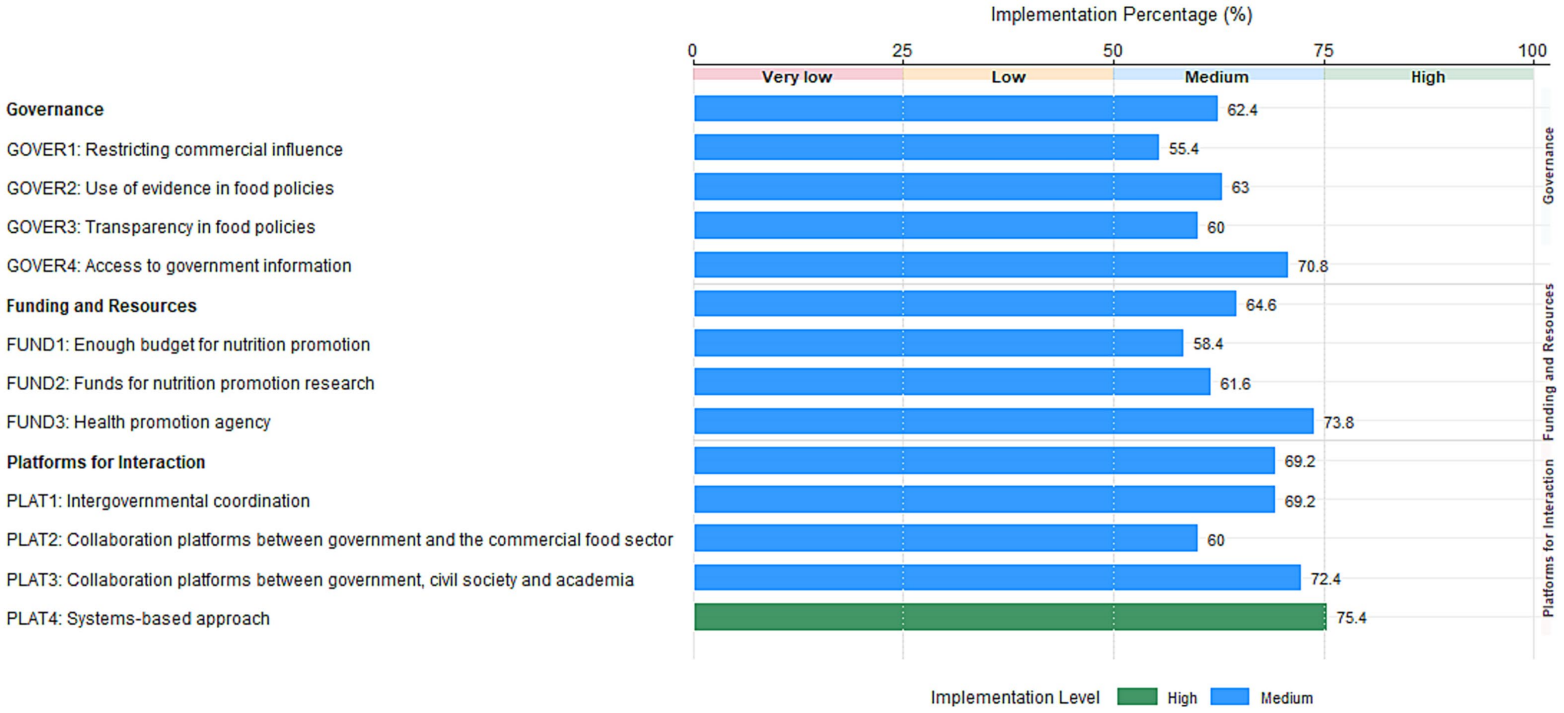
Level of implementation of indicators against international best practices.

Overall, implementation levels of policies across the three domains—governance, funding and resources, and platforms for interaction—were medium (Table 5; Figure 3). Among the 11 indicators, 9.1% demonstrated high levels, while 90.9% showed medium. No indicators exhibited low or very low.

**Table 5:** Score, implementation percentage and level for different indicators.

Domain/Indicator	Score	Implementation percentage	Level^a^
Governance	3.12 ± 0.98	62.4%	Medium
GOVER1: Restricting commercial influence	2.77 ± 0.93	55.4%	Medium
GOVER2: Use of evidence in food policies	3.15 ± 0.99	63.0%	Medium
GOVER3: Transparency in food policies	3.00 ± 1.00	60.0%	Medium
GOVER4: Access to government information	3.54 ± 0.97	70.8%	Medium
Funding and resources	3.23 ± 0.96	64.6%	Medium
FUND1: Enough budget for nutrition promotion	2.92 ± 1.04	58.4%	Medium
FUND2: Funds for nutrition promotion research	3.08 ± 0.76	61.6%	Medium
FUND3: Health promotion agency	3.69 ± 0.95	73.8%	Medium
Platforms for interaction	3.46 ± 0.90	69.2%	Medium
PLAT1: Intergovernmental coordination	3.46 ± 1.05	69.2%	Medium
PLAT2: Collaboration platforms between government and the commercial food sector	3.00 ± 0.91	60.0%	Medium
PLAT3: Collaboration platforms between government, civil society and academia	3.62 ± 0.65	72.4%	Medium
PLAT4: Systems-based approach	3.77 ± 0.83	75.4%	High

^a^Refers to the classification of policy formulation and implementation levels for each indicator and domain based on the implementation percentage: high (≥75%), medium (50–75%), low (25–50%), and very low (<25%).

The governance domain scored 3.12 ± 0.98 (62.4% implementation percentage), with GOVER4 performing best (3.54 ± 0.97, 70.8%). All indicators within this domain were at a medium level. The funding and resources domain scored 3.23 ± 0.96 (64.6% implementation percentage). Significant variation existed among indicators: FUND3 reached 3.69 ± 0.95 (73.8%), while FUND1 was only 2.92 ± 1.04 (58.4%), both falling within the medium range. The platforms for interaction achieved the highest score (3.46 ± 0.90, 69.2%). PLAT4 reached 3.77 ± 0.83 (75.4%), rated as high, while the remaining indicators were at medium levels.

### Governance evaluation

Regarding GOVER1, the Law of the People’s Republic of China on Donations to Public Welfare Causes strictly regulates donation acceptance practices and limits commercial entities’ influence on policy formulation (29). For GOVER 2, the Law of the People’s Republic of China on Food Safety and its implementing regulations establish a risk-based regulatory mechanism (30); the Chinese Dietary Guidelines and the China Food and Nutrition Development Outline implement a regular update system adhering to evidence-based principles (31). Regarding information disclosure, China’s regulatory framework ensures comprehensive information release covering budgets, statistics, and health research findings (32) (GOVER4). However, transparency in the decision-making process remains an area for improvement, particularly in the openness of policy formulation stages (GOVER3).

### Funding and resources evaluation

Regarding budget for nutrition promotion (FUND1), the budget for chronic disease prevention and control accounted for 10.6% of the public health special fund and 0.54% of the overall health budget, both lower than the 12.26% share of diet-related health losses (33). However, cumulative investments exceeding 286 billion yuan have been made in nutrition improvement programs for students and children in impoverished areas (34), benefiting a large population. Regarding research funding (FUND2), overall funding for nutrition-related chronic disease research has increased. Between 2015 and 2019, the National Natural Science Foundation of China funded 1,152 nutrition-related projects totaling approximately 610 million yuan (35). However, overall investment remains insufficient, and there is a lack of major special projects in nutrition. Regarding institutional development (FUND3), China has established multiple national and provincial health promotion organizations, including the China Health Promotion Foundation, the China Health Promotion and Education Association, and the China Student Nutrition and Health Promotion Association, all dedicated to improving population nutrition.

### Platforms for interaction evaluation

At the intergovernmental level (PLAT1), a cross-departmental coordination system has been established through the State Council Inter-Ministerial Joint Conference for the Prevention and Control of Major Diseases, the National Food and Nutrition Advisory Committee, and the National Nutrition and Health Guidance Committee. Collaboration platforms between government and the commercial food sector (PLAT2) operate through standing communication and feedback channels with industry, including the China Food and Drug Enterprise Quality Safety Promotion Association, the China Dairy Association, and the China Beverage Industry Association. Collaboration platforms between government, civil society and academia (PLAT3) are anchored by mass organizations such as trade unions and the Communist Youth League, together with professional societies—the Chinese Nutrition Society, Chinese Preventive Medicine Association, and China Cuisine Association, provide expert support for policy formulation through regular participation mechanisms. Additionally, initiatives such as the National Healthy Lifestyle Campaign, nutrition science outreach programs, comprehensive chronic disease prevention demonstration zones, and health-promoting schools further advance nationwide collaboration between government and local organizations to jointly improve the food environment and public nutrition and health status (PLAT4).

## Discussion

Governance, funding and resources, and platforms for interaction constitute the core components of the infrastructure support dimension within the Food-EPI framework. Existing research indicates that policy ineffectiveness often stems from weak interdepartmental collaboration, uneven fiscal investment, and lagging monitoring system development (36). In recent years, as China advances ecological civilization, rural revitalization, and national nutrition initiatives, it faces practical demands for optimizing resource allocation and strengthening multisectoral coordination (37, 38). Against this backdrop, applying Food-EPI to conduct a systematic evaluation of China’s food and nutrition policies not only helps identify critical shortcomings in the current governance system but also provides empirical evidence and improvement pathways for enhancing overall policy effectiveness and advancing the modernization of nutrition governance.

In this study, most indicators were classified at a “medium” level. On the one hand, this result reflects that China’s food and nutrition policies have established a certain foundation in relevant areas, but there remains room for continuous improvement compared with international best practices. On the other hand, it may also be related to the common central tendency bias in expert scoring (39). When evaluating complex policy systems, experts tend to provide more cautious, moderate judgments and are less likely to use extreme scores (40), especially in situations where policies have been partially established but their implementation effects are not yet sufficient, and where there are regional variations in execution. More importantly, in this study, a “medium” level does not simply indicate an average level of policy performance, but rather reflects a pattern of implementation in which policies are partially in place but have not yet fully realized their intended effects. Specifically, such ratings often correspond to several practical situations: first, relevant policy frameworks have been established, but supporting mechanisms (e.g., financial support, technical capacity, or regulatory systems) remain incomplete; second, policies have achieved some progress in certain regions or population groups, but have not yet reached broad and equitable coverage; and third, existing policy instruments are limited in their intensity or continuity during implementation, resulting in their potential effects not being fully realized. Thus, a “medium” level reflects not only whether policies are in place, but also that their effectiveness and operational maturity remain at a developing stage. This further suggests that the primary challenge has shifted from the absence of policies to the quality of implementation—namely, improving the stability of policy execution, strengthening institutional coordination, and reducing disparities in implementation across regions. In other words, the “medium” status identified in this study essentially reflects that China’s food and nutrition policies are in a transitional stage, shifting from framework establishment toward high-quality implementation.

This study examines the performance of the policy governance in terms of institutional fairness, scientific decision-making, and public communication. Regarding Use of evidence in food policies (GOVER2), while implementation remains at a medium level, several institutionalized mechanisms have been established. For instance, longitudinal datasets from the National Nutrition and Health Survey provide a robust data foundation for dynamically monitoring policy outcomes and identifying intervention gaps. Such localized, sustained data accumulation is a core prerequisite for shifting from experience-driven to evidence-driven approaches. Similar practices are observed in low- and medium-income countries where mathematical modeling supports policy decision-making during public health crises (41). However, research indicates that translating scientific findings into policy applications faces multiple barriers, including institutional fragmentation, political resistance, and limited access to “actionable data” (42). This suggests that data provision alone is insufficient to ensure effective utilization; complementary organizational mechanisms and institutional designs are also needed to bridge the gap between science and policy (43). Although China has relatively strong capacity for data generation, a stable closed-loop mechanism linking “evidence generation – evidence synthesis – policy adoption” has not yet been fully established. This gap is particularly evident in policy contexts involving multiple sectors, where different departments have varying requirements regarding the type, timeliness, and applicability of evidence. As a result, research evidence often cannot be efficiently translated into policy tools that are operationally actionable within administrative systems. Regarding transparency in food policies (GOVER3), this study indicates its implementation remains at a medium level. While some sectors have established information disclosure and public consultation procedures—such as mechanisms for soliciting feedback on policy drafts—transparency coverage remains concentrated primarily during the policy implementation phase. Openness in policy agenda setting and design remains relatively weak. This “front-end closed, back-end open” model limits the public’s ability to influence policy formation early on, thereby undermining policy legitimacy and social acceptance. International comparisons reveal that countries like New Zealand have established more robust legal and practical frameworks safeguarding citizens’ right to information, achieving higher levels of information accessibility (25). Furthermore, research indicates that policy transparency does not automatically translate into public understanding or support; its effectiveness is moderated by the presentation format and level of detail—disclosures that are overly complex or technical may actually reduce comprehension (44). Therefore, enhancing transparency requires not only expanding the scope of information disclosure but also prioritizing readability, accessibility, and interactivity to genuinely achieve informed participation.

In terms of funding and resources, China exhibits sustained growth in research investment but structural imbalances in budget allocation. Regarding funds for nutrition promotion research (FUND2), this study indicates its implementation level is medium. Rapid advancements in food and nutrition-related science and technology provide crucial methodological support for policy implementation and effectiveness evaluation. This is evidenced by sustained funding from institutions like the National Natural Science Foundation for research on nutrition-related chronic diseases such as obesity and metabolic disorders, alongside the overall growth in nutrition-related projects and funding scale in recent years (35). Concurrently, technological advancements in agriculture are driving nutrition-oriented agricultural development (45). The refinement of food testing and traceability systems—such as HACCP and solid-phase extraction—effectively safeguards food safety. These cross-disciplinary innovations collectively form the technological governance for China’s food and nutrition policy implementation. However, structural imbalances persist in aligning funding and resources with disease burden (FUND1). Data indicates that China’s budget for chronic disease prevention and control, as a proportion of both public health special funds and total health expenditures, falls significantly below the relative weight of diet-related risk factors in overall health loss (33). This mismatch not only diminishes the potential effectiveness of policy interventions but also reflects that current fiscal resource allocation has yet to fully recognize the public health priority status of nutrition and health issues. In contrast, the Netherlands has implemented Health Assessment Audits to systematically direct funding toward chronic disease prevention and high-impact areas, offering valuable insights for optimizing resource allocation in China (46).

In terms of platforms for interaction, China has preliminarily established a multi-level, multi-stakeholder policy coordination platform. Results indicate that the implementation of national initiatives such as the National Healthy Lifestyle Campaign and Comprehensive Chronic Disease Prevention and Control Demonstration Zones (PLAT4) has reached a high level, demonstrating phased success in operational coordination mechanisms. The relatively high score of this indicator may be attributed to China’s strong administrative mobilization capacity, pilot-based scaling mechanisms, and vertically integrated organizational system in major public health initiatives. Through special campaigns, demonstration area development, and target-based accountability management, relevant policies can be rapidly transmitted from central-level planning to local-level implementation (9–11). However, the coordination platform faces deep-seated challenges in practice, including coordination mechanisms operating in name only and insufficient implementation effectiveness. Current efforts remain heavily reliant on the health sector as the primary driver. Other relevant departments (such as agriculture, education, and market regulation) and food enterprises exhibit insufficient initiative and sense of responsibility, hindering substantive cross-departmental collaboration throughout the entire policy formulation and implementation process. In contrast, New Zealand (25) has established a national public health nutrition expert panel, effectively ensuring broad cross-departmental and interdisciplinary support at every stage from policy design to implementation. Simultaneously, New Zealand leverages a systematic evaluation mechanism not only to track policy progress but also to encourage sectors like agriculture, economics, and the environment to proactively integrate food environment issues into their own policy agendas. This significantly enhances the overall coordination of food policies. Mexico’s (47) experience further demonstrates that relying solely on the public health sector to drive policy implementation is insufficient. It is essential to establish a robust legal framework that clearly defines cross-departmental responsibilities, ensures multi-stakeholder participation, and simultaneously guides improvements in market behavior. Therefore, the domain of coordination exhibits a pattern characterized by “strong project implementation but weak institutional coordination”. In other words, while coordination can be achieved at the level of specific actions, there are still notable shortcomings in terms of shared responsibility, routine collaboration, and joint decision-making across departments. This also explains why the coordination domain performs relatively well on certain operational indicators, yet has not reached a high level overall.

Overall, the differences observed both across the three domains and among indicators within each domain are mainly driven by three types of factors. First, differences in policy attributes: indicators that are highly operational and can be advanced through targeted campaigns or special initiatives are more likely to achieve higher scores, whereas those involving institutional reform, interest coordination, and long-term resource allocation tend to improve more slowly. Second, differences in governance mechanisms: domains with clearly designated lead agencies, established accountability systems, and vertical implementation pathways are more likely to demonstrate effective execution, while those relying on horizontal coordination among multiple departments are more prone to fragmented responsibilities. Third, differences in resource allocation approaches: project-based support forms such as research programs and pilot initiatives are relatively well-developed, whereas institutionalized investments including dedicated budgets, permanent institutions, and sustained data-sharing mechanisms, remain insufficient. It should be noted that the clustering of indicators within the “medium” category does not imply a single underlying cause, but rather reflects the combined effects of shared structural constraints and domain-specific mechanisms. Across domains, common challenges—including limited implementation capacity, insufficient cross-sectoral coordination, and underdeveloped institutional support systems—generally constrain policy effectiveness. However, the specific manifestations differ by domain. In the governance domain, a “medium” level is more likely to reflect situations where institutional rules are in place but are not fully utilized in decision-making processes, for example, where evidence is generated but not consistently integrated into policy workflows. In the funding and resources domain, it is characterized by increasing levels of investment accompanied by suboptimal allocation structures, that is, “resources are available but not optimally targeted.” In contrast, in the domain of platforms for interaction, it is reflected in the ability to achieve coordination in short-term initiatives, but with limited development of routine and institutionalized cross-sectoral collaboration mechanisms, that is, “coordination exists but lacks sustainability.”

Based on these findings, several targeted recommendations are proposed. First, in response to the issue of misalignment between budget allocation and disease burden in the resource support domain, it is recommended to establish a dedicated population nutrition promotion budget and to develop a dynamic adjustment mechanism linking budget allocation to the burden of chronic diseases. This would help direct more financial resources toward high-burden areas such as food environment improvement, obesity prevention, and diet-related chronic disease control, thereby directly addressing the structural imbalance in resource allocation identified in this study (9, 10). Second, to address the gap between evidence generation and policy adoption in the infrastructure support domain, it is recommended to establish routine mechanisms for translating nutrition policy evidence at both national and provincial levels. Examples include developing interdisciplinary policy advisory platforms and creating evidence synthesis and rapid-response mechanisms to improve the efficiency with which scientific evidence informs policymaking (48). Third, regarding the issue that transparency is concentrated in the later stages of policy implementation, with insufficient upstream participation, it is recommended to shift public consultation and expert engagement earlier into the agenda-setting and policy design stages, while also improving feedback disclosure mechanisms. This would enhance transparency, responsiveness, and public acceptability in the policy development process. In addition, to address the pattern of strong project-based coordination but weak institutional coordination in the coordination domain, it is recommended to further clarify the roles and responsibilities of relevant sectors—including agriculture, education, market regulation, and finance—through institutional design. Establishing formal cross-sector coordination bodies and shared accountability mechanisms would help reduce reliance on a single leading sector, such as health authorities. To tackle the issue of insufficient professional capacity and institutional network support in the resource domain, it is recommended to build a professional nutrition institution network covering both provincial and municipal levels, alongside the development of multidisciplinary training programs to systematically strengthen workforce capacity. Furthermore, in response to the fragmented data systems affecting both infrastructure support and resource domains, it is recommended to integrate nutrition and health data currently dispersed across departments and to establish a national nutrition and health big data platform. This platform should support standardized data sharing and collaborative governance, enabling multi-source data integration and analysis while ensuring data security, thereby providing robust support for evidence-based policymaking, monitoring, and dynamic policy adjustment. Finally, considering the substantial regional differences in economic development, governance capacity, and digital infrastructure, it is recommended that these reforms be implemented through a phased approach of “pilot–evaluation–scale-up.” Initial demonstration projects can be conducted in more developed regions, followed by gradual expansion nationwide, to enhance feasibility and implementation efficiency.

This study has several limitations. First, although all members of the expert panel possessed relevant professional backgrounds in food and nutrition policy, the scoring of indicators was inevitably influenced by individual differences in perception and subjective judgment. In addition, the composition of the expert panel was somewhat unbalanced, with members predominantly from academia, relatively limited representation from industry, and no inclusion of non-governmental organization (NGO) representatives involved in food environment issues. While this composition helped ensure the scientific rigor of policy analysis, it may have limited the diversity of perspectives and affected a comprehensive understanding of policy practicality, industry responses, and mechanisms for social participation. Future studies could further optimize the composition of expert panels by incorporating representatives from NGOs, industry, and other practice-oriented sectors to enhance the representativeness and robustness of evaluation results. Second, this study used an expert scoring approach to assess policy implementation, which may be subject to central tendency bias—that is, scorers tend to choose moderate values rather than extreme ones—resulting in some indicators being concentrated at a “medium” level and reducing the differentiation between categories. To mitigate this issue, in addition to categorical ratings, this study also reports continuous scores, implementation percentages, and standard deviations for each indicator, allowing for a more nuanced reflection of differences across indicators. Future research could incorporate more objective criteria or quantitative evidence to cross-validate expert scoring results. Finally, regarding international comparisons, differences in data availability, indicator definitions, and monitoring methods across countries meant that data from some countries could not be included in the analysis, thereby affecting the breadth and depth of cross-country comparisons to a certain extent. Future research should continue to explore the development and application of standardized evaluation frameworks to improve the international comparability of results.

## Conclusion

Compared with international best practices, China’s food and nutrition policies have established a certain foundation in infrastructure support, resource support, and coordination. However, there remains room for improvement, and overall the system is still in a transitional stage, moving from framework establishment toward high-quality implementation. Going forward, efforts should focus on optimizing resource allocation, strengthening professional support systems, and enhancing data integration and sharing, in order to further improve policy implementation effectiveness and support the modernization of nutrition governance (33).

## Data Availability

The original contributions presented in the study are included in the article/Supplementary material, further inquiries can be directed to the corresponding author.
