# Supplementary material for: Assessment of China’s food and nutrition policies based on healthy food environment policy index (food-EPI): focusing on governance, funding and resources, platforms for interaction
Source: Front Nutr. 2026 May 19;13:1831021. doi: 10.3389/fnut.2026.1831021 (PMC13226163; doi:10.3389/fnut.2026.1831021)
Supplement: Supplementary file 1 [file Table_1.DOCX]

Supplementary Table S1: Domestic and International Policy Evidence on Governance, Funding and Resources, and Platforms for Interaction

| **Domain** | **Indicator** | **International Best Practices** | **Local Evidence** |
| --- | --- | --- | --- |
| Governance | GOVER1: Restricting commercial influence | (1) Australia: The Australian Public Service Commission must adhere to codes of conduct governing conflicts of interest, public-private partnerships, and lobbying.  (2) United States: Mandatory lobbying registration systems exist at both federal and state levels, requiring disclosure of financial information and imposing strict penalties. | The Law of the People's Republic of China on Donations to Public Welfare Causes stipulates that only public welfare associations and public institutions may accept donations. The government may only accept donations under extremely exceptional circumstances, such as natural disasters or at the request of foreign entities, and may not itself be a beneficiary. |
|  | GOVER2: Use of evidence in food policies | Australia: The National Health and Medical Research Council (NHMRC) develops evidence-based guidelines through a nine-step process mandated by law, implementing a mechanism of five-year updates and mandatory ten-year reviews. | (1) the Law of the People’s Republic of China on Food Safety stipulates that where risk assessments confirm the unsafety of food-related products, national regulatory authorities must take corresponding measures in accordance with the law.  (2) The Dietary Guidelines for Chinese Residents establish a mechanism for periodic revision every 5 to 10 years, updated in reference to international standards and evidence evaluation systems.  (3) The formulation and updating of the China Food and Nutrition Development Outline and its supporting policies both adhere to the principle of evidence-based decision-making. |
|  | GOVER3: Transparency in food policies | Australia/New Zealand: The Food Standards Australia New Zealand Act 1991 requires Food Standards Australia New Zealand (FSANZ) to publicly engage all relevant stakeholders when developing new standards and to adhere to the principle of transparency. | (1) The Implementing Regulations of the Law of the People’s Republic of China on Food Safety explicitly stipulate that public consultation must be conducted when formulating national food safety standards plans and programs.  (2) Certain policies (such as the Rules for Nutrition Labeling of Food Items in the Catering Industry) release drafts for public comment prior to implementation to solicit societal feedback.  (3) However, in practice, many policies only disclose their implementation processes, while their formulation stages still lack sufficient transparency. |
|  | GOVER4: Access to government information | Australia/New Zealand: The Freedom of Information Act in Australia and New Zealand grants the public a legal right to access documents held by government departments and most agencies, thereby promoting greater government transparency and openness. | (1) The Regulations of the People's Republic of China on Open Government Information explicitly stipulate that governments at all levels must proactively disclose information including development plans, fiscal budgets, statistical data, major projects, and outcomes of various supervisory inspections.  (2) Numerous institutions have established governance for information disclosure, such as the Administrative Measures for Information Disclosure by Healthcare Service Units, the Administrative Measures for Information Disclosure by the National Natural Science Foundation of China, and the Administrative Measures for Information Disclosure by the Chinese Academy of Sciences. These documents clarify specific operational procedures and responsibilities for information disclosure.  (3) The China National Nutrition and Health Survey Report is regularly released by the State Council Information Office. The public may access it through publications or the official website of the Chinese Center for Disease Control and Prevention.  (4) The National Survey on Students' Physical Fitness and Health is conducted every 5 to 6 years and publicly released to society. |
| Funding and Resources | FUND1: Enough budget for nutrition promotion | (1) New Zealand: The “Healthy Eating, Healthy Action” program implemented in 2008-2009 received approximately US$67 million in funding, accounting for 0.6% of the total health budget, while dietary risk factors contributed to 11.4% of health losses.  (2) Thailand: In 2012, local government expenditures on nutrition and health care totaled 29.4345 billion baht (approximately US$840 million), accounting for 7.57% of total health expenditures—a tenfold increase from 2011 levels. During the same period, health losses attributable to dietary risk factors constituted approximately 10% of the total. | (1) There exists a significant gap between China's chronic disease prevention and control budget and its investment in nutrition and health. Currently, the budget for chronic disease prevention and control accounts for only 10.6% of the total budget for public health special tasks and 0.54% of the total health and wellness budget, far below the 12.26% share of dietary risk factors in health losses. Although the budget includes items such as nutrition monitoring, it does not allocate dedicated funds for nutrition.  (2) Concurrently, China has continuously increased investments in nutrition improvement for impoverished regions. From 2011 to 2021, the central government allocated a cumulative total of 196.434 billion yuan to the Rural Compulsory Education Student Nutrition Improvement Program, benefiting over 33.6 million students across 137,000 schools. From 2012 to 2017, 90 billion yuan was invested in implementing the Child Nutrition Improvement Project in impoverished areas. By 2023, this initiative had cumulatively benefited 19.28 million infants and young children in regions that had achieved poverty alleviation. |
|  | FUND2: Funds for nutrition promotion research | (1) Australia: The National Health and Medical Research Council Act mandates the identification of National Health Priority Areas (NHPA). For 2015-2016, obesity, diabetes, and cardiovascular health were designated as the three priority areas.  (2) Thailand: The National Research Council increased funding for obesity and diet-related chronic disease research to approximately 37.87 million baht in 2014, representing a sixfold increase from the 2013 allocation of approximately 6.88 million baht. | (1) Documents such as the Medium- and Long-term Plan for the Prevention and Treatment of Chronic Diseases in China and the Maternal and Child Safety Action Plan (2018–2020) explicitly require the implementation of departmental responsibilities and fiscal investment, strengthening government leadership, and enhancing financial and service support for impoverished regions.  (2) While the number and funding of research projects on nutrition-related chronic diseases in China show an overall upward trend, the total investment remains insufficient, and there is a lack of major special projects specifically targeting nutrition. The National Natural Science Foundation of China has prioritized nutrition-related chronic diseases such as cardiovascular and cerebrovascular diseases and obesity. From 2015 to 2019, it funded a total of 1,152 projects in the nutrition field, amounting to approximately 610 million yuan. Human nutrition is a funding priority, yet few key projects were approved during this period. The National Science and Technology Plan included human nutrition research in its priority areas during the 12th Five-Year Plan period. During the 13th Five-Year Plan period, it was not directly included, with only one project related to maternal and child nutrition under the “Special Program for Basic Scientific Research.” While no major special program was established for nutrition, it was addressed within other key programs such as diabetes research. |
|  | FUND3: Health promotion agency | Australia: The Victorian Health Promotion Foundation (VicHealth) in Australia is the world's first health promotion foundation. It was established by the state parliament through the Tobacco Act 1987, which clearly defined the foundation's statutory objectives. | China has formulated relevant plans to promote the establishment of multiple health promotion institutions aimed at improving population nutrition, including national and provincial-level health promotion organizations such as the China Health Promotion Foundation, the China Health Promotion and Education Association, and the China Student Nutrition and Health Promotion Association. |
| Platforms for Interaction | PLAT1: Intergovernmental coordination | (1) Finland: The Finnish National Nutrition Council, operating under the Ministry of Agriculture and Forestry, is an intergovernmental expert body composed of representatives from multiple sectors. It is responsible for providing advisory, coordination, and monitoring services in nutrition and food-related fields.  (2) Malta: Established under the Healthy Lifestyle Promotion and Care of NCDs Act 2016, the Interministerial Advisory Committee on Healthy Lifestyles advises on policies concerning physical activity, nutrition, and chronic disease prevention and control. | (1) The State Council Inter-Ministerial Joint Conference on Major Disease Prevention and Control comprises 30 departments and units. It is responsible for platforms for interaction in national major disease prevention and control, formulating key prevention and control policies, and resolving critical issues.  (2) The National Food and Nutrition Advisory Committee was established in 1993 under the Ministry of Agriculture. Comprising experts from multiple fields, it undertakes research, advisory services, and knowledge dissemination in food and nutrition.  (3) The National Nutrition and Health Guidance Committee was jointly established in 2019 by 18 departments including the National Health Commission. Its purpose is to establish a departmental cooperation mechanism and coordinate the implementation of the National Nutrition Plan. |
|  | PLAT2: Collaboration platforms between government and the commercial food sector | United Kingdom: Between 2011 and 2015, the UK implemented the “Responsibility Deal,” encouraging voluntary commitments from food companies to engage in chronic disease prevention and control. Chaired by the Secretary of State for Health, the initiative brought together food businesses, non-governmental organizations, and public health bodies. A dedicated working group was established to drive the implementation of specific projects. | China has established communication and feedback mechanisms with the food industry through industry associations such as the China Food and Drug Enterprise Quality and Safety Promotion Association, the China Dairy Association, and the China Beverage Industry Association, to collaboratively advance the implementation of healthy food policies. |
|  | PLAT3: Collaboration platforms between government, civil society and academia | Brazil: The National Council of Food and Nutrition Security (CONSEA) is an advisory body on food and nutrition security policy directly under the Office of the President. Its membership comprises one-third government officials and two-thirds non-governmental representatives. This body is responsible for formulating and promoting public policies that ensure citizens have access to healthy diets. It maintains corresponding institutions at the state and municipal levels, creating a multi-tiered consultation system. Although Congress theoretically has the power to overturn its proposals, CONSEA wields significant policy influence in practice due to its constitutional status and broad social representation. | (1) The “Healthy China 2030” Planning Outline calls for establishing cross-departmental coordination and accountability mechanisms, while mobilizing social organizations to jointly advance the Healthy China initiative.  (2) Multiple civil society groups, including the Chinese Nutrition Society, the Chinese Preventive Medicine Association, and the China Cuisine Association, maintain regular and constructive interactions with the government. |
|  | PLAT4: Systems-based approach | (1) New Zealand: Led by the Ministry of Health, “Healthy Families NZ” is a large-scale community chronic disease prevention initiative covering 10 regions and benefiting over one million people.  (2) Australia: The “Healthy Together Victoria” program, jointly funded by state and federal governments, aims to promote healthy lifestyles across all life settings and address the root causes of health issues through comprehensive policies. | (1) National Healthy Lifestyle Initiative: Launched in 2007, this initiative has progressed through two phases—“Healthy 121” and “Three Reductions, Three Healths”—aiming to mobilize social forces and promote healthy living. By 2017, it had reached over 80% of counties (districts) nationwide.  (2) Annual nutrition awareness campaigns—including National Nutrition Week, National Food Safety Awareness Week, “May 20th” China Student Nutrition Day, and “May 15th” National Iodine Deficiency Disorders Prevention Day—have been conducted nationwide for many years.  (3) Comprehensive Chronic Disease Prevention and Control Demonstration Zones: To address the chronic disease burden, China began establishing county-level demonstration zones in 2009. By June 2020, 488 national-level demonstration zones had been established.  (4) Health-Promoting Schools: Introduced in China in 1995, this concept emphasizes collaborative efforts among governments, schools, and communities to create safe, healthy environments and provide comprehensive health promotion for students and community members. |
